# Inducing and blocking the goal to belong in an experimental setting: goal disengagement research using Cyberball

**DOI:** 10.1007/s11031-022-09975-w

**Published:** 2022-09-05

**Authors:** Farina Rühs, Werner Greve, Cathleen Kappes

**Affiliations:** Institute of Psychology, University of Hildesheim, Universitätsplatz 1, D-31141 Hildesheim, Germany

**Keywords:** Goal disengagement, Goal induction, Goal blockage, Social exclusion, Self-regulation

## Abstract

In the present research, the Cyberball ostracism paradigm was adapted for experimental goal disengagement (GD) research: the goal to belong to a particular group is first induced in participants (via social interaction) and then blocked (via social exclusion) to trigger GD processes. In an online group setting, we experimentally tested the procedure’s suitability to investigate goal disengagement processes. A pilot study demonstrated successful induction of the goal to belong. In the main study (*N* = 180), exclusion from the group reduced participants’ perceived goal attainability (indicating goal blockage) and desirability (indicating goal disengagement) and their well-being. Regarding the regulatory functions of GD, results were mixed. During work on individual tasks, goal desirability decreased further and well-being was largely restored. However, GD changes were correlated only with changes in negative affect (and not other well-being measures). Findings suggest the procedure’s suitability for studying GD experimentally and employing it to investigate other measures of GD processes and their functionality in more detail.

## Introduction

People often encounter obstacles in the pursuit of their goals. Extensive knowledge exists on the conditions for successful and persistent goal pursuit despite such obstacles (for a recent review see, e.g., Brandstätter & Bernecker, 2022). However, less research has investigated how people disengage from unattainable goals without limiting their well-being, thereby maintaining their ability to act. According to several theories of developmental regulation (e.g., the two-process model of developmental regulation: Brandtstädter & Rothermund, 2002, or motivational theory of lifespan development: Heckhausen et al., 2010), the ability to let go of a blocked goal altogether or at least devalue it (goal disengagement) predicts individual well-being and quality of life (meta-analysis: Barlow et al., 2020). Yet, previous studies particularly addressing goal disengagement have been mainly observational and/or cross-sectional with a focus on individual differences in the capacity to disengage. These (non-experimental) studies thus allow little insight into the causal processes underlying goal disengagement as a functional response to a blocked goal.

For this, experimental designs are needed, in which (1) commitment to a sufficiently self-relevant goal is induced in a first step and (2) this newly activated goal is then experimentally blocked. This also allows for (3) the assessment of situational goal disengagement processes (instead of the capacity to react with such processes in general).

**Fig. 1:**
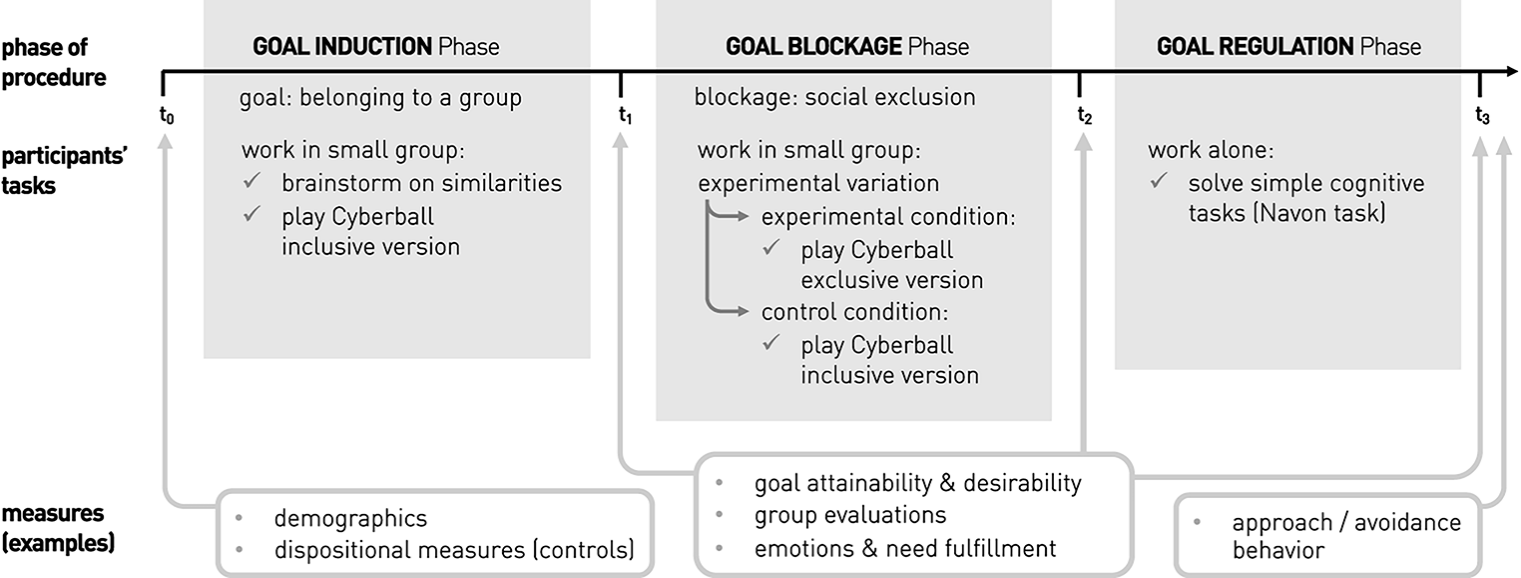
*Overview of the experimental procedure*

The present research reports findings on the suitability of a newly adapted procedure that meets these requirements. The proposed procedure is based on the Cyberball paradigm (Williams & Jarvis, 2006), a virtual ball-tossing game where co-players’ behavior can be preprogrammed, for example, to include or exclude the participant. In this way, the goal to belong to a particular group can first be induced in participants (via social interaction) and then blocked (via social exclusion; see Fig. 1 for an overview of the procedure). This provides opportunities to examine the processes of goal disengagement causally and in more detail.

## Inducing and blocking goals in experimental settings

### Goal induction

People’s expectations and motives when choosing and prioritizing goals are assumed to influence the processes of goal disengagement if goals are blocked (e.g., Ntoumanis & Sedikides, 2018; Ntoumanis et al., 2014). However, confounding of these variables cannot be controlled for in observational designs where participants introspect about self-selected goals. Thus, approaches that experimentally induce goals are necessary to avoid these confounds.

Earlier experimental research often used one of two forms of goal induction. On the one hand, a number of studies used various forms of goal priming (for an overview, see, e.g., Moskowitz & Gesundheit, 2009). On the other hand, several studies had participants work on performance tasks under the assumption that participants were motivated, and thus pursued the goal to do their best on the task (e.g., Aspinwall & Richter, 1999; Henderson et al., 2007; Koppe & Rothermund, 2016; Lench & Levine, 2008).

However, both forms of goal induction have limitations with respect to the study of goal disengagement. In priming approaches it is hard to say whether classical priming really activates goals or some other mental representations that do not classify as such (Förster et al., 2007). For approaches that use performance tasks, the assumption that dedicated work on a task always implies the presence of a performance goal is a separate empirical question that has not yet been sufficiently explored. For both approaches, it is of particular importance that only the blockage of reasonably desirable and self-relevant goals should trigger self-regulatory processes (like goal disengagement) to a quantitative and temporal extent that can be realistically measured in a laboratory setting regarding changes over time.

The proposed procedure in the present research addresses these limitations. First, it focuses on a goal that is assumed to be sufficiently self-relevant and experimentally malleable at the same time: the goal to belong to a newly formed, anonymous group. Second, the activation of the goal is measured explicitly.

Many different conceptions of goals and thus also criteria about what constitutes a goal exist (for an overview see, e.g., Austin & Vancouver, 1996; Brandstätter & Hennecke, 2018). However, there is broad consensus on the assumption that goals can be defined as “internal representations of desired states, where states are broadly construed as outcomes, events or processes”, and these “internal represented desired states range from biological set points for internal processes […] to complex cognitive depictions of desired outcomes” (Austin & Vancouver, 1996, p. 338). Most researchers addressing goals would further agree that, in order for a representation to qualify as a goal, the desired state should be principally attainable and that there should be a certain degree of engagement for reaching this state. Thus, in line with classical expectancy-value approaches to goals (e.g., Ajzen & Kruglanski, 2019; Hollenbeck & Klein, 1987), in the proposed procedure, successful goal activation should at least be reflected in participants having an internal representation of belonging to the group as a desired state, i.e., they should assign importance to that goal (desirability). Moreover, they should also consider the goal to be reasonably achievable (attainability).

In the proposed procedure, the activation of the goal to belong to a new, anonymous group is achieved by putting participants through a group membership induction procedure adapted from social psychological studies. Research using the minimal group paradigm showed that identification with an arbitrarily assigned new group and own-group favoritism can be induced even in anonymous contexts and with only minimal cues (e.g., Otten, 2016; Pinter & Greenwald, 2011; Tajfel et al., 1971). Whether this induction also goes along with activation of the goal to belong to this group, to our knowledge, has not yet been empirically investigated and is one question of the present research. Theoretically, however, hierarchical models of motivation (e.g., Elliot & Church, 1997) make a strong case for this. The need to belong is thought to be an universal and fundamental human motive or need (Baumeister & Leary, 1995). According to hierarchical models of motivation, goals are “midlevel constructs” that are “structurally situated between global motivational dispositions and specific behaviors” (Elliot & Church, 1997, p. 219). They are “concrete manifestations” (Elliot & Church, 1997, p. 219) of the higher order motivational dispositions and regulate behavior in accordance with them. That is, in a situation with opportunities to realize a particular motive/need (in the proposed procedure: a situation in which people interact in groups, as an opportunity to satisfy the need to belong), people should develop goals (in the proposed procedure: the goal to belong to the assigned group) that regulate behavior in the direction that makes satisfaction of the need more likely.

### Goal blockage

Developmental regulation theories (e.g., Brandtstädter & Rothermund, 2002) assume that perceived blockage of a goal is one condition that can trigger disengagement processes from this goal (such as decreased goal desirability as one aspect of cognitive-affective disengagement, see below). A perceived blockage should be reflected in reduced subjective goal attainability. Here again, observational studies cannot rule out confounding of goal disengagement processes with the subjective evaluation of attainability (or blockage) of a goal. Moreover, when using retrospective assessment of stress and coping responses after a goal blockage in non-longitudinal designs, memory biases associated with goal regulation processes could occur (Kensinger & Ford, 2020). Even in studies that experimentally induce performance goals, knowledge and beliefs about one’s abilities could influence not only the subjective attainability and desirability of that goal, but also the perception of and response to obstacles in the pursuit of that goal (e.g., Ajzen & Kruglanski, 2019; Burnette et al., 2013). Hence, in order to causally investigate goal disengagement as a reaction to goal blockage, studies should experimentally vary goal blockage and examine its consequences regarding goal disengagement prospectively. Unfortunately, such studies are rare (for designs with an experimental manipulation of goal blockage or goal difficulty, see, e.g., Aspinwall & Richter, 1999; Ntoumanis, Healy, Sedikides, Duda et al., 2014; for a prospective longitudinal approach without experimental goal blockage, see, e.g., Bermeitinger et al., 2018).

The proposed procedure in the present research addresses this gap. Blockage of the goal to belong to the group is manipulated via exclusion of the participant from a previously formed group in a virtual ball-tossing game (control condition: inclusion in the game). The blockage should be reflected in a decline in perceived attainability of the goal (note that we do not expect attainability to drop to zero, as participants do not know whether they will have further opportunities to interact during the course of the study). Moreover, such experiences of social exclusion have been shown to be associated with negative consequences for the individual’s well-being (e.g., affect and self-esteem; Gerber & Wheeler 2009; Leary, 2015), even in the anonymous experimental setting applied in Cyberball (Hartgerink et al., 2015). In the proposed procedure, they thus should represent a plausible cause for regulatory processes to occur.

## Goal disengagement as a regulatory response to blocked goals

Several theories of developmental regulation (e.g., Brandtstädter & Rothermund, 2002; Heckhausen et al., 2010) make assumptions about how people react when their current situation deviates from their desired state (goal). Although they differ in detail, the different proposed processes broadly can be assigned to two modes of regulation: goal engagement and goal disengagement (review: Wrosch, 2011; theoretical and empirical integration: Haase et al., 2013). While goal engagement refers to processes aimed at continuing goal pursuit despite challenges, goal disengagement refers to processes of adaptation of goals to given circumstances and letting go of blocked goals.

Presumably, the goal to belong to a newly formed anonymous group is moderately self-relevant, but not important enough to require holding on to it at all costs. Therefore, we assume that for most participants in the proposed procedure the blockage of the goal to belong to this group will trigger goal disengagement processes (although individual differences in dispositions such as “coping styles” will explain some variation, e.g., Heckhausen & Wrosch, 2016). This is also supported by the finding that reduced perceived attainability of a goal is associated with increased occurrences of an action crisis (a phase of conflict between holding on and letting go of a goal) that in turn is assumed to be one antecedent or aspect of disengagement processes (Ghassemi et al., 2017).

### Goal disengagement: functions and processes

Regarding goal disengagement, cognitive-affective disengagement (i.e., reduced psychological commitment) can be distinguished from behavioral disengagement (i.e., reduced effort, see Wrosch et al., 2003; see also Brandstätter & Bernecker 2022). How cognitive-affective and behavioral disengagement relate to each other is open to theoretical discussion and empirical investigation. For example, there seem to be goals from which one (temporarily) disengages behaviorally, but which remain relevant on the cognitive-affective level (“frozen goals”, Davydenko et al., 2019; “goal shelving”, Mayer & Freund, 2021).

However, developmental goal regulation theories (e.g. Brandtstädter & Rothermund, 2002) highlight the importance of “inner” psychological distancing from the goal, i.e. cognitive-affective disengagement processes (e.g., devaluing the goal, reducing its importance / the “psychological commitment”, emotional detachment). These processes are assumed to play a “crucial role” for the adaptive (relieving) function of goal disengagement in situations of goal blockage (Brandstätter & Bernecker, 2022, p. 3.19, see also Brandtstädter 2007; Wrosch et al., 2013). Thus, the present research focusses primarily on processes of cognitive-affective goal disengagement. In the proposed procedure, these disengagement processes are expected to be associated with restoring well-being after being excluded from a group. Findings of a recent meta-analysis (Barlow et al., 2020) suggest that this functionality of the disengagement processes may occur particularly in terms of reducing negative compared to enhancing positive affect.

One model that makes explicit assumptions about the information-processing underlying disengagement processes following goal blockage is the two-process model of developmental regulation (e.g., Brandtstädter & Rothermund, 2002). It assumes that goal disengagement processes “reset the cognitive system” (Brandtstädter & Rothermund, 2002, p. 123) in a way, that the hedonic difference between the current situation (here: experience of rejection) and the desired state (here: belong to the group) can be reduced on the side of the desired state. One possible way to achieve this is to devalue the goal as a whole. In the context of the proposed procedure (cognitive-affective) goal disengagement thus should, among other things, be reflected by reduced subjective desirability of the goal to belong to the group. Moreover, the two-process model assumes that disengagement relates to a mind-set characterized by (among other things) more holistic processing, broader attention and increased availability of cognitions that facilitate disengagement from the goal. Thus, a further indicator of such sub-processes of disengagement could be the devaluation of specific aspects of the goal (e.g., devaluing the group: “that group is not so nice, I don’t have to belong to it”). Furthermore, if cognitive-affective disengagement is accompanied by behavioral disengagement as well, this could become visible in behavioral deprioritization (e.g. fewer actions to reconnect to the former group or more actions to connect to another group).

To be sure, we do not claim to capture all facets of disengagement from the goal to belong in the present study. Rather, the devaluation processes examined in the present research are intended as initial starting points for testing a newly adapted paradigm by investigating one highly relevant aspect of goal disengagement (from the point of view of the developmental regulation theories mentioned above). On this basis, further research can hopefully be conducted on various other possible sub-processes of disengagement by introducing other measures into the paradigm.

### Goal disengagement: Assessment

Assessment of goal disengagement processes and their outcomes is challenging for several reasons. First, we know almost nothing about the time scales on which these processes take place. Their duration will certainly depend on several factors and, hence, will vary between persons and situations. Second, these processes are arguably at least partly inaccessible to introspection and thus to self-report (Brandtstädter & Rothermund, 2002). Thus, a deeper understanding of goal disengagement processes requires studies in which cognitive-affective and behavioral changes as indicators of goal disengagement are examined over time and in relation to a specific (blocked) goal.

A few such longitudinal and goal-specific studies exist. Some studies captured goal disengagement processes during phases of struggle in goal thriving as change in cognitions and appraisals regarding a specific goal such as its desirability and attainability (Brandstätter et al., 2013; Brandstätter & Herrmann, 2016; Brandstätter & Schüler, 2013; Ghassemi et al., 2017). Other studies applied situational, goal-specific adaptations of existing dispositional measures in situations of real-life goal blockages (Thompson et al., 2011, 2013). Still other studies used behavioral indicators for goal disengagement such as task-switching behavior when working on unsolvable tasks (Kappes & Thomsen, 2020; Koppe & Rothermund, 2016; Thomsen et al., 2017; van Randenborgh et al., 2010).

While these studies have taken important new approaches using situational assessment of goal disengagement and thus provided new insights into the processes of goal disengagement, they suffer in part from the problems with observational studies already discussed and the question of whether “giving up” behaviorally in experimentally given tasks is the same as goal disengagement (e.g., Barber et al., 2012). The present research addresses these issues by using both repeated self-report and behavioral measures in an experimental design to investigate whether they di- or converge and how they relate to well-being.

## The present research

Based on theoretical considerations and empirical results presented above, we adapted the Cyberball Paradigm (Williams & Jarvis, 2006) for investigating goal disengagement. In the adapted procedure six participants at a time take part in an online group study, forming two anonymous groups (they work with pseudonyms via chat and cannot see each other). They then go through 3 phases:

1. *Induction phase*: The goal to belong to a particular group is induced in all participants. Participants are randomly assigned to two groups, learn the pseudonyms of their group members and in their group work collaboratively on two tasks: a chat-based brainstorming on similarities (actual participants work together in real time) and playing Cyberball (inclusive version: alleged virtual teammates are preprogrammed to include the participants).
2. *Blocking phase (experimental manipulation)*: The goal to belong to the group is blocked by the experience of social rejection in half of the participants (experimental condition). In their assigned group, they play Cyberball again, but this time they are excluded by the (alleged) virtual teammates. Participants in the control condition also play Cyberball in their assigned group again, but in the inclusive version, so that no goal blockage occurs.
3. *Regulation phase*: Participants individually work on simple cognitive tasks to give possible goal regulation processes some time to occur.

The present research aimed at answering two main questions of validating the proposed procedure: Can playing Cyberball in the exclusive version cause a perceived blockage of attaining the goal to belong to a particular group? And if there is a blockage: Do participants respond to this blockage with cognitive-affective disengagement (i.e., decreased subjective desirability) of the goal to belong? Moreover, initial attempts were made to make possible (sub-)processes of cognitive-affective goal disengagement (changes in cognitive representations / evaluations) and behavioral goal disengagement (behavioral deprioritization) empirically accessible. In doing so, the interrelationships of the indicators of goal disengagement with each other and with well-being could be examined in more detail.

Study Design, Hypotheses and Analysis Plan were preregistered prior to data collection on OSF.^1^ Also further details on all measured variables and the implementation and supplementary materials are uploaded on OSF.

## Design and hypotheses^2^

Participants went through the full procedure shown in Fig. 1. This resulted in a 2 (goal blockage: no vs. yes, between) × 3 (time: after induction phase (t1) vs. after blocking phase (t2) vs. after regulation phase (t3), within) mixed design.

### Hypotheses regarding goal induction and goal blockage (H1 to H3)

We expected that participants would develop the goal to belong to their group during the induction phase (t0 to t1). If this was the case, mean goal desirability and attainability ratings after the induction phase should be similar to those of a pilot study that experimentally tested the manipulation (described below). Moreover, as a more indirect indicator of goal activation and in line with social-identity-research (e.g., Everett et al., 2015), we expected an own-group favoritism effect in explicit group evaluations at t1 (**H1**).

If the procedure was successful in manipulating the goal blockage, goal attainability should decrease during the blocking phase (t1 to t2) in participants in the blockage condition, but not in the control condition (**H2**). Moreover, as social exclusion in Cyberball is a painful experience (Hartgerink et al., 2015), this should become visible in a decline in excluded (but not included) participants’ well-being (measured via self-reports on emotions and needs as it is common in ostracism research, see, e.g., Williams, 2009) from t1 to t2 (**H3**).

### Hypotheses regarding regulatory responses induced by goal blockage (H4 to H6)

If the procedure was successful in eliciting (cognitive-affective) goal disengagement processes through goal blocking, the desirability of the goal to belong to the particular group should decrease in participants in the blockage condition after exclusion from t2 to t3 (**H4**).

Moreover, based on theoretical considerations regarding possible (sub-)processes of goal disengagement (e.g., Brandtstädter & Rothermund, 2002), we also investigated two more indicators of such processes: (1) the devaluation of one’s own group compared to the other group (measured via change in explicit group evaluations in the regulation phase) and (2) approach behavior towards former out-group and avoidance behavior towards former in-group members (measured through the number of ball tosses while playing Cyberball a third time after the regulation phase, this time with one former in-group and one former out-group member). We expected a decrease of the own-group bias during the regulation phase (t2 to t3, **H5**) and a behavioral deprioritization, assessed as a behavioral approach/avoidance tendency which is more in favor of former out-group members at the end of the regulation phase (t3, **H6**) for participants in the blockage condition (but not for those in the control condition).

### Hypotheses regarding the functionality of assumed regulatory responses (H7 to H10)

If the used indicators (change in goal desirability, change in group evaluation and approach/avoidance behavior) indeed capture functional regulation responses to goal blockage, participants in the blockage condition should also recover from the stress of being excluded during the regulation phase. This should be reflected in an improvement of well-being (i.e., increase of positive affect and need fulfillment and decrease of negative affect) during the regulation phase (t2 to t3) in subjects in the blockage condition compared to the control condition (**H7**). Moreover, this positive change also should be associated with the assumed regulatory responses at the same time, that is, with changes in goal desirability and explicit group evaluations. More precisely, a stronger decrease of goal desirability should be associated with a stronger increase of positive affect and need fulfillment and decrease of negative affect (**H8**). Moreover, the more the own-group bias decreases, the higher the increase of positive affect and need fulfillment and decrease of negative affect should be (**H9**). Lastly, the behavioral approach/avoidance tendency towards former in- and out-group members at the end of the regulation phase (t3) should be associated with well-being at the same time. More precisely: The more in favor of former out-group members the approach/avoidance-tendency is, the higher the positive affect and need fulfillment and the lower the negative affect should be for subjects in the blockage condition (**H10**).

## Method

### Participants

Participation was open to all German-speaking people born between 1996 and 2002 who had access to an Internet-enabled device with a keyboard. The study was advertised online via various social media channels, private contacts and the digital board for study announcements of the conducting institute. Sample size planning was based on an a priori power analysis for testing within-between-interaction effects in a repeated measures ANOVAs with 3 measurements and 2 groups using G*Power 3.1 (Faul et al., 2009). Since comparable studies to estimate effect sizes were not available for all dependent variables, we only roughly assumed an effect of medium size (*f* = 0.25). Further specifications were a power near to .85, an alpha error of .05 and a total sample size divisible by six. This resulted in a planned sample size of 180 participants (power = .86).

During study implementation, some participants did not show up and for some sessions not all places could be assigned (missing persons were replaced with confederates). Thus, 35 sessions were performed in April and May 2021 until 183 persons had participated. Three persons were excluded from the data analysis because their data could not be unambiguously assigned due to errors in the individual code, resulting in a final sample of *N* = 180 (140 females, *M*_age_ = 22.24, *SD*_age_ = 1.98). Regarding employment status, 116 participants were university students, 30 salaried employees or civil servants and 28 pupils or trainees (one person was currently searching for work and five people chose other or did not respond to this question). Participants were distributed equally across conditions (*n* = 90 in each condition); mean age and gender distribution did not differ statistically significantly between conditions.

### Procedure^3^

To avoid expectation effects, we used the cover story that the study investigated creativity in anonymous online groups. Participants were informed in advance by email about the study procedure and completed an initial online questionnaire regarding demographic data and dispositional control variables. In the synchronous part six participants logged into a virtual study room using an assigned ID (cameras and microphones were disabled for everyone except the study director, communication was only possible via chat) and were then randomly assigned one of six pseudonyms (Ulut, Ocla, Ydai, Ipit, Delcha, Palup).^4^

Within the virtual study room, all information was presented to the participants via both audio and slides and tasks were performed using links to external sites. All participants completed exactly the same tasks; only in the blocking phase was there an experimental variation between the conditions. Participants were randomly assigned to either the no blockage (control) or blockage (experimental) condition^5^. As compensation, participants received 12 € in the form of a voucher (Amazon or Thalia) or a donation on their behalf to Primaklima e.V.

#### Tasks in the induction phase

After a general introduction, all participants were told that they would form two anonymous groups. They had one minute to learn the group membership presented on a slide. After this, participants had to brainstorm together with their previously assigned group members on similarities (what they have in common as a group of people born around the turn of the millennium) for 5 min. Lastly, they played an inclusive version of Cyberball (by clicking per mouse on the person they wanted to pass the ball to next) seemingly again with their two group members. Co-players were programmed so that each player got the ball equally often. The play ended after a total of 30 throws (after about two minutes). To keep the cover story going and to intensify the experience, instructions emphasized that it was not about execution in the ball game per se, but about mentally visualizing the entire experience.

To ensure that the goal to belong to a particular group was actually induced by this procedure, we conducted a pilot study.^6^ In this online group-study, 126 participants were randomly assigned to three different experimental conditions assumed to elicit varying degrees of goal activation. All participants took part in a brainstorming task and played an online ball-game. However, each of the two tasks could be completed either in the assigned group (brainstorming: find commonalities; ball game: play Cyberball with player preprogrammed to include the participant) or individually (brainstorming: find uses of a brick; ball game: play individual game against a virtual wall). The condition in which both tasks were done in the group (group condition: intended implementation in the paradigm) was contrasted with two control conditions concerning goal attainability and desirability: the condition in which both tasks were completed alone (individual condition) and a mixed condition (one of the tasks was done alone, the other in the group). One-way ANOVAs revealed that the experimental manipulation had a statistically significant effect of medium size on both belonging goal desirability, *F*(2, 123) = 5.17, *p* = .007, ω^2^ = .06; and attainability, *F*(2, 123) = 8.39, *p* < .001, ω^2^ = .11, in the assumed direction. Compared to those who did both tasks alone, participants who did both tasks in the group rated the goal to belong to the group as more desirable, *M*_group_ = 64.05 (*SD* = 22.58), *M*_individual_ = 43.99 (*SD* = 26.64), *M*_Diff_= 20.07, 95% CI [4.96; 35.18], *t*(59.97) = 3.19, *p*_tukey_= .006, and attainable, *M*_group_ = 51.33 (*SD* = 27.64), *M*_individual_ = 26.23 (25.82), *M*_Diff_= 25.01, 95% CI [8.60; 41.42], *t*(57.72) = 3.67, *p*_tukey_= .002.

#### Tasks in the blocking phase (experimental variation)

In the second step, participants played Cyberball again. For participants in the control condition, all settings remained identical to those in the induction phase (no goal blockage). In the experimental condition, however, participants were excluded from the game after two initial throws, that is, they did not get the ball from the (alleged) team members anymore (goal blockage). The number of throws and time played were equal between conditions: 30 throws in about 2 min.

#### Tasks in the Regulation Phase

In the third step, all participants worked individually on a variant of the Navon task (Navon, 1977) implemented via PsyToolkit (Stoet, 2010, 2017). Participants were presented with figures that have global and local features: the whole figure looks like a letter (e.g., like an “L”, global feature) but is made up of small letters (e.g., “O”s, local features). Figures containing different global and local letters are used as stimuli and participants have to decide via pressing two different keys if a specific letter (here “H” or “O”) is present in the figure or not (regardless of global or local). In total, the participants completed 50 such trials (duration: about three minutes).

#### Measures

All dependent measures except behavioral approach/avoidance tendency were administered three times (once after each phase of the procedure) via an online questionnaire that was generated by SoSci Survey (Leiner, 2021). At each measurement time point, the variables were collected in the following order: subjective goal desirability and attainability, group evaluations, emotions and need fulfillment and distractor items related to creativity to keep the cover story going. Means, standard deviations and reliability coefficients of all dependent variables measured via self-report are displayed in Table 1, separated according to time of measurement. Additionally, at t3, the behavioral approach/avoidance tendency was measured following the questionnaire via playing a different version of Cyberball again (see below). For all dependent variables that were measured more than once, two indices of change (from t1 to t2 and from t2 to t3) were calculated in addition to the indices for the individual measurement time points. Here, the preceding value was always subtracted from the following one, so that positive change values represent an increase and negative change values represent a decrease of the respective variable over the two measurement points considered.

**Table 1:** Means, standard deviations, reliabilities, and two-way ANOVA statistics for repeated study variables

Measure			no blockage		blockage			ANOVA										
	time	α */ r*^a^	*M*	*SD*	*M*	*SD*		Effect	Hypothesis		*F*		*df*		*p*		${{\upomega }}^{2}$	
bga																		
	t1	.72	60.12	25.08	60.62	22.60		C			16.63		1,178		**< .001**		.04	
	t2	.92	61.38	26.96	36.93	28.03		T			33.59^b^		1.55, 275.60		**< .001**		.04	
	t3	.95	57.80	29.32	39.32	26.16		C × T	H2b		32.18^b^		1.55, 275.60		**< .001**		.04	
na																		
	t1	.63	1.21	0.34	1.25	0.36		G			70.60		1,178		**< .001**		.16	
	t2	.94	1.29	0.59	2.58	1.23		T			60.61^b^		1.90, 338.69		**< .001**		.14	
	t3	.94	1.17	0.40	1.82	0.97		C × T	H3b, H7b		47.18^b^		1.90, 338.69		**< .001**		.11	
pa																		
	t1	.79	3.94	0.69	3.89	0.69		C			18.02		1, 178		**< .001**		.05	
	t2	.89	3.74	0.90	2.81	0.93		T			63.25		2, 356		**< .001**		.09	
	t3	.85	3.80	0.81	3.47	0.82		C × T	H3b, H7b		31.02		2, 356		**< .001**		.05	
nf																		
	t1	.91	3.90	0.63	4.00	0.49		C			58.42		1,177		**< .001**		.14	
	t2	.98	3.74	0.81	2.19	0.99		T			134.46^b^		1.86, 328.5		**< .001**		.23	
	t3	.95	3.82	0.63	3.25	0.85		C × T	H3b, H7b		93.48^b^		1.86, 328.5		**< .001**		.17	
bgd																		
	t1	.79	59.38	28.53	62.41	25.51		C			3.30		1,178		.071		.01	
	t2	.90	60.35	30.62	53.04	24.80		T			34.13^b^		1.73, 307.69		**< .001**		.04	
	t3	.94	55.33	32.42	39.38	25.95		C × T	H4b		16.04^b^		1.73, 307.69		**< .001**		.02	
rge																		
	t1	−	0.92	1.34	0.87	1.35		C			52.64		1,178		**< .001**		.13	
	t2	−	0.92	1.36	−1.51	2.24		T			49.07^b^		1.48, 263.98		**< .001**		.09	
	t3	−	0.71	1.27	−0.92	1.87		C × T	H5b		43.99		1.48, 263.98		**< 0.001**		0.08	

*Note.* bga = belonging goal attainability; bgd = belonging goal desirability; na = negative affect; pa = positive affect; nf = need fulfillment; rge = relative group evaluation; no blockage = control condition; blockage = experimental condition; C = condition of experimental variation (no blockage vs. blockage); T = time of measurement. Statistically significant (*p* < .05) results are in bold

^a^ For scales consisting of two items (bga and bgd) Pearson’s *r* was used as an estimate of reliability, for all other scales Cronbach’s α is reported

^b^ Mauchly’s test indicated that the assumption of sphericity was violated (*p* < .05), Greenhouse-Geisser correction was applied

#### Subjective goal attainability and desirability

For two group membership related goals (“belong to my group”, “be liked by my group”) participants each rated the importance and the attainability of that goal during the study, on a slider (visual analog scale) from *difficult to attain* / *not at all important* (coded as zero) to *easily attainable* / *extremely important* (coded as 100). The mean of the two importance and attainability ratings, respectively, was calculated, resulting in one measure each of *belonging goal attainability* and *belonging goal desirability*.

#### Explicit group evaluations

Evaluations of the own group and the other group were captured using three positive and negative statements each (examples: “I feel sympathy for my group / the other group”, “I feel dislike for my group / the other group”). Participants indicated how much these applied to them (answers on a five-point scale from 1 = *not at all* to 5 = *extremely*). For further analyses, evaluations were aggregated in three steps: First, means were calculated separately for evaluations of the own and the other group and each valence, forming four indices for each participant (*own-positive*, *own-negative*, *other-positive*, *other-negative*). Second, negative and positive evaluations were combined in one evaluative index for each group (formula: *own evaluation* = *own*-*positive* – *own*-*negative, other evaluation* = *other*-*positive* – *other*-*negative*). Third, these indices where combined again into one aggregated evaluative index reflecting the relative evaluation of the own vs. the other group (formula: *relative group evaluation* = *own* – *other*). Here, positive values mean that the own group is evaluated better than the other group (i.e., own-group favoritism).

#### Behavioral approach/avoidance tendency

At t3, all participants played Cyberball again in the inclusive version (same settings as in the induction phase), but this time (seemingly) with one person from their own group and one person from the other group. Because the game consists of 30 throws and is programmed so that all players throw equally often, this means that participants could pass the ball to another player nine to ten times. Each time they had the choice to throw the ball to a player from their own group or to the new player from the other group. The throws were recorded and an index was created: behavioral approach/avoidance tendency = passes to other group member / total passes of subject. Values above 0.5 represent a preference for the player from the other group and thus behavioral deprioritization, values below 0.5 represent a preference for the member of the own group.

#### Emotions and need fulfillment

For each of six positive (cheerful, happy, relaxed, interested, attentive, determined) and six negative (upset, angry, downhearted, sad, afraid, shaky) emotions, participants indicated how strongly they experienced this emotion at the moment (answers on a five-point scale from 1 = *not at all* to 5 = *extremely*). For further analyses, one index representing *positive affect* and one representing *negative affect* was derived for each participant by calculating the mean of the related items.

Participants completed a German version of Williams’s (2009) 20-item need-threat-questionnaire that is widely used in ostracism-research, because it addresses needs that are especially relevant in social interactions (and can be threatened by social exclusion and promoted by inclusion). In relation to four superordinate needs (*belonging*, *self-esteem*, *control*, *meaningful existence*) five statements are presented in each case describing the extent to which the respective need is fulfilled at the moment (examples belonging: “I feel I belong to the group”; self-esteem: “I feel liked”; meaningful existence: “I feel invisible” (reverse coded), control: “I feel powerful”). Participants indicated how much these statements applied to them (answers on a five-point scale from 1 = *not at all* to 5 = *extremely*). For further analyses, an overall composite index was calculated (subscales were not considered in this study).

In order to make it clear to which time period the statements on emotions and need satisfaction refer, the introductions to the items and the time form of the items itself were slightly adjusted depending on the measurement time point. At t1 and t2, participants were asked how they felt during the last ball game and the items were presented in the past tense. At t3, however, participants were asked about how they were currently feeling and the items were presented in the present tense.

#### Analysis plan

All analyses were performed using JASP (JASP Team, 2021). In a first step, descriptive statistics of main study variables (separated by condition and time of measurement where appropriate) were calculated and distributions were checked for deviations from normality. For hypotheses tested via simple group comparisons or bivariate correlations, detailed presentation is omitted here (see the preregistration for details), instead the results are reported directly in the respective results sections (this applies to hypotheses 1, 6, 8, 9, and 10). The changes of the respective measures postulated in hypotheses 2, 3, 4, 5 and 7 all were tested in two ways: First based on condition-dependent intraindividual changes and second in terms of differences between conditions at different measurement time points. Even though different outcome variables were examined in the different hypotheses, the analytic logic remained the same. For all analyses regarding differences in intraindividual change, one-sided independent *t* tests were performed, using the experimental condition (goal blockage: yes vs. no) as independent variable and the change variable addressed in the hypothesis as dependent variable (see Table 2 for an overview of tests and how they correspond to the formulated hypotheses). For all analyses regarding differences between conditions at different measurement time points, two-factorial ANOVAs with repeated measures on one factor (time of measurement: after the induction phase vs. after the blocking phase vs. after the regulation phase, between factor: blockage vs. no blockage) were calculated (see Table 1 for an overview of all six ANOVAs, the outcome variables applied and how they correspond to the formulated hypotheses). A statistically significant interaction effect was expected if the respective hypothesis was true. Subsequently, planned contrasts were calculated to test the specific assumptions about comparisons between conditions at measurement time points contained in the hypotheses. This involved contrasting the experimental with the control condition at each measurement time point individually (at t1: C1, at t2: C2, at t3: C3) and additionally testing the interaction from t2 to t3 (i.e., whether the condition differences between t2 and t3 were different: C4, see Table 3 for an overview of calculated contrasts and how they correspond to the formulated hypotheses, see Supplementary Table 1 in the osf for a matrix showing the contrast coding). All results were considered to be statistically significant when *p* < .05.^7^

**Table 2:** Means, standard deviations, and *t*-test statistics for change variables and behavioral approach/avoidance tendency

	no blockage		blockage		*t* test				
Measure	*M*	*SD*	*M*	*SD*	Hypothesis	*t*	*df*	*p*	*d*
bga t1–t2	1.26	17.80	−23.69	29.57	H2a (μ_nb_ > μ_b_)	6.86^a^	178	**< .001**	1.02
bga t2–t3	−3.58	14.89	2.39	14.66	add. (μ_nb_ ≠ μ_b_)	−2.71^b^	178	**.007**	−0.40
na t1–t2	0.08	0.51	1.34	1.26	H3a (μ_nb_ < μ_b_)	−8.78^a^	178	**< .001**	−1.31
na t2–t3	−0.11	0.65	−0.76	0.94	H7a (μ_nb_ > μ_b_)	5.38^a^	178	**< .001**	0.80
pa t1–t2	−0.20	0.60	−1.08	0.99	H3a (μ_nb_ > μ_b_)	7.22^a^	178	**< .001**	1.08
pa t2–t3	0.06	0.65	0.66	0.88	H7a (μ_nb_ < μ_b_)	−5.21^a^	178	**< .001**	−0.78
nf t1–t2	−0.16	0.70	−1.81	1.09	H3a (μ_nb_ > μ_b_)	12.04^a^	177	**< .001**	1.80
nf t2–t3	0.08	0.56	1.06	0.87	H7a (μ_nb_ < μ_b_)	−9.00 ^a^	178	**< .001**	−1.34
bgd t1–t2	0.97	16.06	−9.37	24.97	add. (μ_nb_ ≠ μ_b_)	3.30	178	**.001**	0.49
bgd t2–t3	−5.02	15.71	−13.66	22.45	H4a (μ_nb_ > μ_b_)	2.99^a^	178	**.002**	0.45
rge t1–t2	0.00	1.53	−2.38	2.61	add. (μ_nb_ ≠ μ_b_)	7.47^a^	178	**< .001**	1.11
rge t2–t3	−0.21	0.95	0.59	1.45	H5a (μ_nb_ > μ_b_)	−4.38^a^	178	−^c^	−
baat	0.49	0.14	0.55	0.17	H6 (μ_nb_ < μ_b_)	−2.39^b^	177	**.009**	−0.36

*Note*. bga = belonging goal attainability; bgd = belonging goal desirability; na = negative affect; pa = positive affect; nf = need fulfillment; rge = relative group evaluation; baat = behavioral approach/avoidance tendency; no blockage = control condition; blockage = experimental condition. Statistically significant (*p* < .05) results are in bold

^a^ Shapiro-Wilk test suggested deviation from normality and Levene’s test non equal variances; however more robust Mann-Whitney and Welch test yielded same results regarding decision for rejecting the null-hypothesis, thus statistics for Student *t* test are reported

^b^ Shapiro-Wilk test suggested deviation from normality; however more robust Mann-Whitney test yielded same results regarding decision for rejecting the null-hypothesis, thus statistics for Student *t* test are reported

^c^ One-sided significance test was not performed because mean difference was in the opposite direction as hypothesized

**Table 3:** Results of contrasts following two-way ANOVAs

Measure			95% CI					
Contrast	Hypothesis	Estimate	*LL*	*UL*	*SE*	*df*	*t*	*p*
bga								
C1	H2b	− 0.50	−7.52	6.52	3.56	178	−0.14	.888
C2	H2b	24.45	16.36	32.54	4.10	178	5.96	**< .001**
C3	add.	18.48	10.30	26.65	4.14	178	4.46	**< .001**
C4	add.	5.97	1.63	10.32	2.20	178	2.71	**.007**
na								
C1	H3b	−0.04	−0.14	0.07	0.05	178	−0.74	.460
C2	H3b	−1.30	−1.58	−1.02	0.14	178	−9.08	**< .001**
C3	H7b	−0.65	−0.87	−0.43	0.11	178	−5.87	**< .001**
C4	H7b	−0.65	−0.89	−0.41	0.12	178	−5.38	**< .001**
pa								
C1	H3b	0.04	−0.16	0.25	0.10	178	0.43	.665
C2	H3b	0.92	0.65	1.19	0.14	178	6.74	**< .001**
C3	H7b	0.33	0.09	0.56	0.12	178	2.67	**.008**
C4	H7b	0.60	0.37	0.83	0.11	178	5.21	**< .001**
nf								
C1	H3b	−0.10	−0.26	0.07	0.08	177	−1.16	.250
C2	H3b	1.55	1.28	1.81	0.14	177	11.43	**< .001**
C3	H7b	0.57	0.35	0.79	0.11	177	5.09	**< .001**
C4	H7b	0.98	0.76	1.19	0.11	177	8.91	**< .001**
bgd								
C1	H4b	−3.02	−10.98	4.94	4.03	178	−0.75	.455
C2	H4b	7.31	−0.89	15.51	4.15	178	1.76	.080
C3	H4b	15.96	7.32	24.59	4.38	178	3.65	**< .001**
C4	H4b	−8.64	−14.35	−2.94	2.89	178	−2.99	**.003**
rge								
C1	H5b	0.05	−0.34	0.45	0.20	178	0.26	.796
C2	H5b	2.43	1.88	2.98	0.28	178	8.79	**< .001**
C3	H5b	1.63	1.16	2.10	0.24	178	6.83	**< .001**
C4	H5b	0.80	0.44	1.16	0.18	178	4.38	**< .001**

*Note.* bga = belonging goal attainability; bgd = belonging goal desirability; na = negative affect; pa = positive affect; nf = need fulfillment; rge = relative group evaluation; C1 = blockage yes vs. no at t1; C2 = blockage yes vs. no at t2; C3 = blockage yes vs. no at t3; C4 = difference between blockage effect at t2 and t3. Statistically significant (*p* < .05) results are in bold

## Results

### Effectiveness of goal induction and experimental goal blockage (H1 to H3)

As predicted, participants (regardless of experimental condition) evaluated their own group statistically significantly more positive than the other group at the end of the induction phase, *M*_own_t1_ = 2.32 (1.16), *M*_other_t1_ = 1.42 (1.18), *t*(179) = 8.92, *p* < .001, *d* = 0.67 (**H1**).^8^ Moreover, means of goal desirability and attainability for all participants were descriptively rather similar to those in the group condition of the pilot study (see method-section for results of the pilot study and Table 1 for results of the main study).

**Fig. 2:**
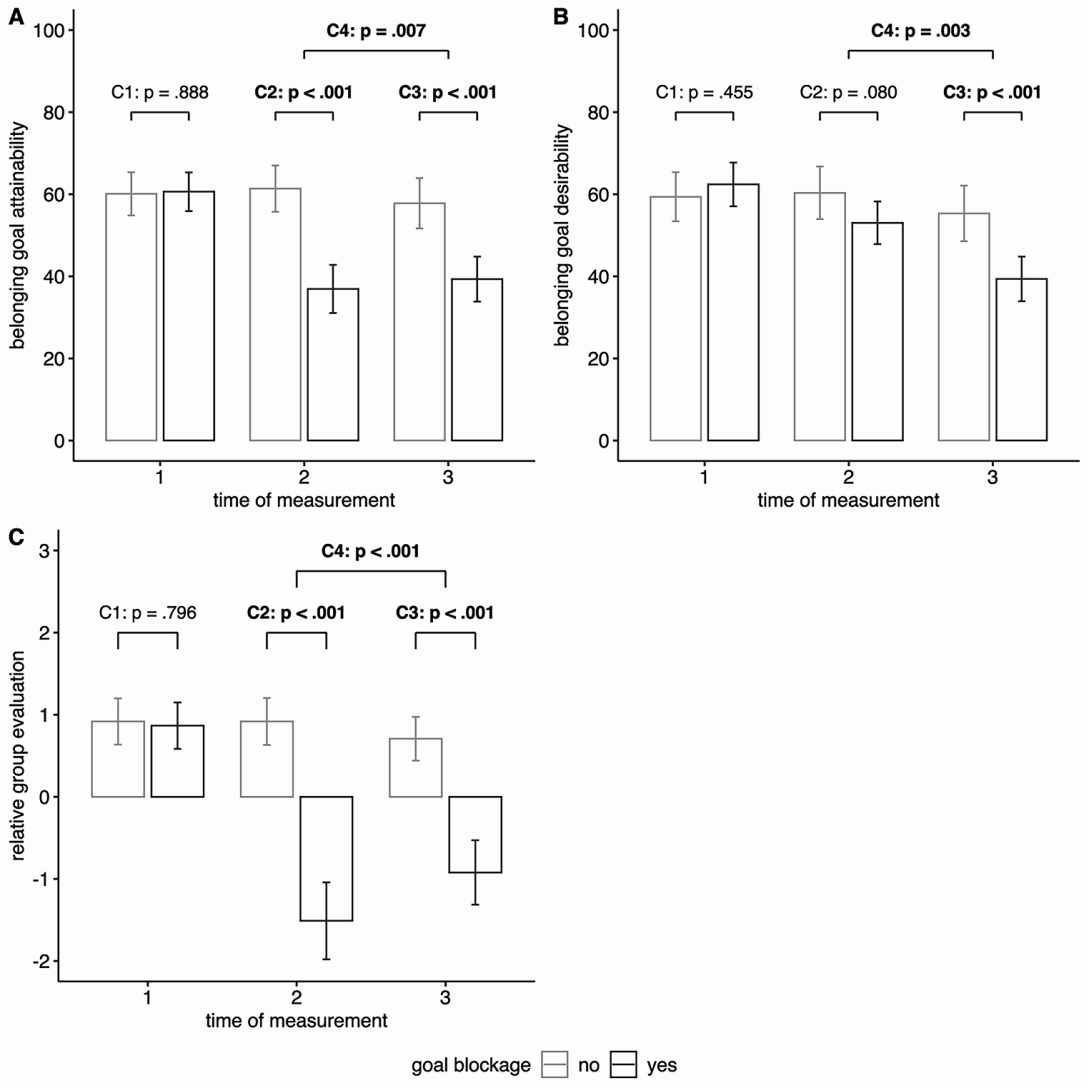
*Goal attainability and indicators of cognitive-affective goal disengagement depending on experimental condition (goal blockage: no vs. yes)* *Note*: Plots A–C show results for different measures **(A)** belonging goal attainability **(B)** belonging goal desirability **(C)** relative group evaluation. Error bars represent 95% confidence intervals of the respective mean. *p*-values are presented for planned contrasts (see also Table 3). Statistically significant (*p* < .05) results are in bold.

Regarding goal blockage, the participants’ perception of belonging goal attainability during the study was examined (**H2**). As predicted, participants in the blockage condition experienced a decrease in goal attainability from t1 to t2, no change was evident in the control condition without blockage. This difference in attainability change between conditions from t1 to t2 was statistically significant (H2a, see Table 2 for detailed results). This pattern was also found at the level of group differences at different measurement time points, whereby participants who were excluded in playing Cyberball in the blocking phase reported statistically significant lower goal attainability at t2 than those who were included in the game. There was a statistically significant interaction effect (condition × measurement time point) in the ANOVA and planned contrasts also showed statistically significant group differences at t2 and t3 but not at t1 (H2b, see Tables 1 and 3 for detailed results and Fig. 2 A).

**Fig. 3:**
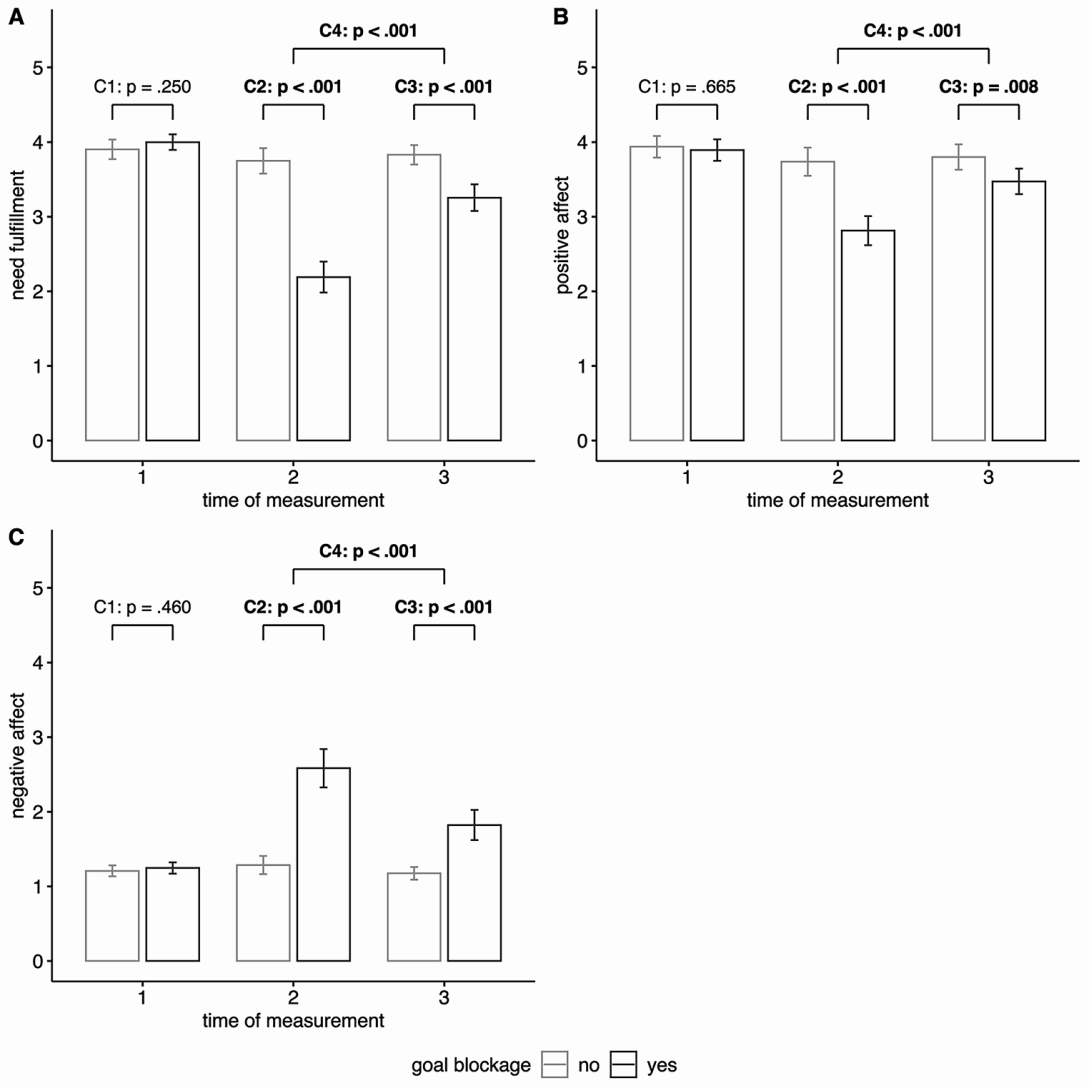
*Indicators of well-being depending on experimental condition (goal blockage: no vs. yes)* *Note*: Plots A–C show results for different measures **(A)** need fulfillment **(B)** positive affect **(C)** negative affect. Error bars represent 95% confidence intervals of the respective mean. *p*-values are presented for planned contrasts (see also Table 3). Statistically significant (*p* < .05) results are in bold.

We also examined affect and need fulfillment in the first phases of the procedure to test whether goal blockage had a negative impact on well-being (**H3**). Results showed the predicted pattern: Participants in the blockage condition experienced a decrease in positive affect and need fulfillment and an increase in negative affect from t1 to t2, whereas, again, there were no substantial changes for participants in the control condition. Differences between conditions in affective change and need fulfillment change from t1 to t2 were statistically significant (H3a, see Table 2 for detailed results). Again, these findings could be supported at the level of group differences at different measurement time points. Whereas there were no group differences at t1, at t2 participants in the blockage condition reported lower positive affect and need fulfillment and higher negative affect compared to those in the control condition. In all three ANOVAs the interaction effect was statistically significant and planned contrasts also showed statistically significant group differences at t2 but not at t1 for all three indicators used (H3b, see Tables 1 and 3 for detailed results and Fig. 3 A–C).

### Regulatory responses Induced by goal blockage (H4 to H6)

We examined change in the desirability of the goal to belong as reflecting (cognitive-affective) goal disengagement. We expected participants in the blockage condition to devalue the goal to belong in the regulation phase (**H4**). Results fit this hypothesis. From t2 to t3, participants in the blockage condition reported a decrease of belonging goal desirability and this decrease was statistically significantly larger than for participants in the control condition, who showed only a small decline during this time (H4a, see Table 2 for detailed results). In the ANOVA, the interaction (condition × time of measurement) proved significant and at t3, participants in the blockage condition reported statistically significant lower goal desirability compared to those in the control condition. Descriptively, conditions also differed regarding goal desirability at t2, but this difference did not reach statistical significance. Moreover, the difference at t2 was statistically significantly smaller than that at t3 (H4b, see Tables 1 and 3 for detailed results and Fig. 2B).

As we considered changes in the relative evaluation of the own-group compared to the other group to be a potential sub-process of cognitive-affective goal disengagement, we expected the same pattern for relative group evaluation as for goal desirability (**H5**). However, results deviated from this hypothesis. While the change in relative group evaluation differed between experimental conditions both from t1 to t2 and from t2 to t3 (see Table 2 for details), there was NO more pronounced decline in the own group preference from t2 to t3 in participants in the blockage condition compared to those in the control condition (H5a). Instead, for participants in the blockage condition, there was a strong decline in relative group evaluation already from t1 to t2 but, contrary to predictions, from t2 to t3 there was a partial recovery from this decline (i.e., the intermediate strong preference of the other group over the own group became weaker again; for participants in the control group no or only small changes occurred). The findings were supported by the ANOVA and planned contrasts, showing statistically significant group differences regarding relative group evaluation at t2 and t3, both in the direction of preference of the other group over the own group, but, different than expected, the difference was greater at t2 than at t3, although even at t3 an own-group favoritism effect was not established again (H5b, see Tables 1 and 3 for detailed results and Fig. 2 C).

The possible indicator of behavioral goal disengagement addressed in this study was the behavioral approach/avoidance tendency at the end of the regulation phase (**H6**). As expected, compared to participants in the control condition, participants in the blockage condition statistically significantly favored a member of the other over their own group in passing the ball in the last Cyberball game at t3. While players in the control condition passed nearly 50% of all their throws to the player of the other group and thus showed no preference, players in the blockage condition threw 55% of their throws to the member of the other group (see Table 2 for detailed results).

### Functionality of assumed regulatory responses (H7 to H10)

We also examined if the measured goal disengagement processes were functional in helping to reduce negative effects of the experience of goal blockage through social exclusion. So, in a first step, we tested whether the participants in the blockage condition would recover in the regulation phase from the stress of the exclusion (**H7**). Actually, compared to the control condition, participants in the blockage condition reported a stronger increase of positive affect and need fulfillment as well as a stronger decrease of negative affect from t2 to t3 (H7a, see Table 2 for detailed results). Nevertheless, well-being was not restored completely in participants in the blockage condition. At t3, statistically significant differences remained between conditions, but (as expected) these were substantially smaller than at t2 (H7b, see Tables 1 and 3 for detailed results and Fig. 3 A–C).

While there was partial recovery of well-being from t2 to t3 in participants in the blockage condition, the change in the three indicator variables (positive affect, need fulfillment and negative affect) was only for negative affect weakly associated with the change in goal desirability at the same time. When goal desirability declined from t2 to t3, negative affect also declined during that time, *r*_s_ = 0.24, *p* = .021^9^ (**H8**, for correlations between all change-variables, see Table 4,^10^). The hypothesis regarding functionality of group revaluation processes (**H9**) was not tested, because tests of Hypothesis 5 showed deviations from the expected pattern and thus the hypothesis based on it had to be rejected. Also, regarding the functionality of approach behavior towards members of the other group at the end of the regulation phase (**H10**), no association could be found with the three indicators of well-being measured at t3 (all *p* > .15).

**Table 4:** Bivariate correlations (*r*_s_) of change variables and behavioral approach/avoidance tendency

Measure	1		2		3		4		5		6		7		8		9		10		11		12		13	
1. bga t1–t2			−.15		−.16		.23	^*^	.17		−.21		.24	^*^	−.26	^*^	.18		.03		.32	^**^	−.13		.05	
2. bga t2–t3	−.35	^***^			−.01		.15		.09		.11		.09		.06		.04		.21	^*^	−.14		.09		−.01	
3. na t1–t2	−.68	^***^	.18				−.41	^***^	−.26	^*^	.26	^*^	−.22	^*^	.30	^**^	.03		−.13		−.09		.03		−.03	
4. na t2–t3	.15		−.25	^*^	−.49	^***^			.25	^*^	−.32	^***^	.13		−.27	^**^	.07		.12		.06		−.09		.09	
5. p.a. t1–t2	.52	^***^	−.14		−.66	^***^	.41	^***^			−.56	^***^	.40	^***^	−.17		.22	^*^	.13		.25	^*^	.12		−.09	
6. p.a. t2–t3	−.15		.24	^*^	.38	^***^	−.66	^***^	−.59	^***^			−.10		.32	^**^	.04		−.16		−.17		.15		.01	
7. nf t1–t2	.68	^***^	−.16		−.76	^***^	.34	^**^	.72	^***^	−.37	^***^			−.38	^***^	−.01		.18		.32	^**^	−.04		−.25	*
8. nf t2–t3	−.21	^*^	.28	^**^	.22	^*^	−.47	^***^	−.38	^***^	.51	^***^	−.52	^***^			−.02		.08		−.16		.30	^**^	.05	
9. bgd t1–t2	.32	^**^	.11		−.14		−.10		.18		−.04		.19		.08				−.02		.07		.01		.08	
10. bgd t2–t3	.44	^***^	−.07		−.40	^***^	.24	^*^	.35	^***^	−.17		.44	^***^	−.10		−.08				−.01		.12		−.11	
11. rge t1–t2	.61	^***^	−.26	^*^	−.70	^***^	.36	^***^	.65	^***^	−.38	^***^	.65	^***^	−.27		.17		.33	^***^			−.40	^***^	−.10	
12. rge t2–t3	−.21		.35	^***^	.36	^***^	−.53	^***^	−.27	^***^	.34	^***^	−.24	^*^	.34	^**^	.13		−.19		−.54	^***^			−.14	
13. baat	.11		−.01		−.14		.04		.11		.04		.09		.05		−.06		.04		.17		−.13			

*Note.* Correlations (Spearman’s rho) are presented separately by experimental condition (blockage (= experimental) condition: below diagonal, *n* = 90; no blockage (= control) condition: above diagonal, *n* = 89 to 90). bga = belonging goal attainability; bgd = belonging goal desirability; na = negative affect; pa = positive affect; nf = need fulfillment; rge = relative group evaluation; baat = behavioral approach/avoidance tendency

^*^p < .05, ^**^p < .01, ^***^p < .001

### Further exploratory analyses

As results regarding the synchronous associations of change in goal desirability in the blockage condition did not show the expected pattern, lagged associations were investigated exploratorily. The results (see Table 4) suggest that change in desirability from t2 to t3 was more strongly associated with antecedent changes in affect and needs than with simultaneous changes: If there was an increase in negative affect or a decrease in positive affect or need satisfaction from t1 to t2, then there was also a decrease in goal desirability from t2 to t3 (medium effect sizes: .35 ≤ |*r*_s_| ≤ .44). But change in goal desirability from t1 to t2 was not associated with any (synchronous or asynchronous) change variable other than synchronous change in goal attainability.

Moreover, changes in relative group evaluations also showed interesting associations with other change variables for participants in the blockage condition (see again Table 4). For both t1 to t2 and t2 to t3, a change in relative group evaluation towards the preference of the other group (relative devaluation of the own group) was synchronously associated with an increase in negative affect and a decrease in positive affect and need satisfaction (large effect sizes for t1 to t2: .65 ≤ |*r*_s_| ≤ .70; medium effect sizes for t2 to t3: .34 ≤ |*r*_s_| ≤ .53). This pattern is contrary to the relationship hypothesized in H9, that a relative group devaluation after the goal blockage would be functional for restoring well-being. Interestingly, changes in relative group evaluation from t1 to t2 and from t2 to t3 were negatively associated. Combined with the descriptive mean values in Table 1 it seems that participants in the blockage condition who relatively devalued their own group more strongly from t1 to t2, also had a stronger reversal of this devaluation from t2 to t3.

## Discussion

### Is the proposed procedure suitable to induce and block goals experimentally?

The main goal of this study was to examine whether the Cyberball paradigm (Williams & Jarvis, 2006) can be applied in such a way that it can be used to investigate causes and effects as well as diachronic dynamics of goal disengagement experimentally. A necessary prerequisite for this is to successfully induce a sufficiently self-relevant goal in the laboratory and to block it afterwards. Results of the present study (combined with the pilot study described in the method section) suggest that the proposed procedure is indeed able to meet these requirements.

At the end of the induction phase participants rated their own group significantly more positive than the other group. This replicates the own-group favoritism effect found in the pilot study. Also, these results are in line with findings from social identity research in computer-mediated communication that increasing the salience of social identity (e.g., via working on common tasks and highlighting commonalities) fosters group affiliation, especially in visual anonymous (“deindividuated”) settings (e.g., Lea et al., 2001; Reicher et al., 1995). Moreover, mean goal desirability and attainability ratings at the end of the induction phase in the present study and in the pilot study indicate a moderate level that could be interpreted as somewhat important and reasonably attainable – and thus indicating some degree of goal activation (in line with classical expectancy-value approaches to goals, e.g., Ajzen & Kruglanski, 2019; Hollenbeck & Klein, 1987). These values seem realistic for a goal artificially induced in a laboratory. Taken together, the results extend findings from social identity research by suggesting that a combination of a group brainstorming and an inclusive Cyberball task can also activate the explicit goal to belong to a particular group.

Most importantly, results of the present study show that playing Cyberball in the exclusive version in the second phase of the study led to a perceived blockage of this goal. Participants in the blockage condition not only reported a decline in well-being after the experience of exclusion (consistent with classic findings on the effects of ostracism by Cyberball, e.g., Hartgerink et al., 2015) but also a reduced subjective attainability of the goal to belong that remained low (but substantially higher than zero) throughout the study.

Against the background of the large variety of different goal types in goal (regulation) research (for an overview see, e.g., Brandstätter & Hennecke, 2018), the question arises whether results of the present study can be generalized to other goal types. Two possible distinguishing criteria of goal types seem particularly relevant when goal disengagement processes are focused: self-relevance and time scale. Classical work in developmental regulation research has particularly examined longer-term personal goals with relatively high self-relevance (see, e.g., Heckhausen et al., 2010). Such goals cannot be induced and blocked in a laboratory setting. Nevertheless, the goal to belong to a particular group can be considered a sufficient approximation (as it relates to basic human needs, causes real pain when blocked, and does occur in real life), albeit on a shorter time scale. Transferability is also supported by findings of research on persistence in goal pursuit, which are similar in laboratory studies with shorter-term goals and in interventions that address longer-term goals (for a review, see Brandstätter & Bernecker, 2022). However, it remains an open research question how comparable goals and their regulatory processes (especially regarding disengagement) are on different time scales (see also Milyavskaya & Werner, 2018).

### Does the proposed procedure induce goal disengagement processes?

Beyond testing the suitability of the procedure for goal induction and blockage, the present study investigated if participants respond to such a blockage with disengagement from the goal to belong. In accord with developmental regulation theories, we focused on cognitive-affective goal disengagement processes as a potential functional regulatory response. Indeed, in the present study the blockage of the goal to belong to a group caused participants to internally disengage from it as indicated by reduction of goal desirability from t2 to t3 (and also already from t1 to t2). This is in line with findings from developmental regulation research, that people disengage when opportunities for goal attainment are low (for an overview, see e.g., Heckhausen et al., 2010).

However, with respect to whether the reduction of goal desirability in the present study was functional in terms of restoring well-being, results were mixed. Overall, participants in the blockage condition showed an extensive but not complete recovery from the negative effects of exclusion after they performed the cognitive task individually. This is consistent with assumptions of Williams’s (2009) temporal need-threat model of reactions to ostracism and studies showing that recovery from ostracism through Cyberball can take some time depending on vulnerabilities (Zadro et al., 2006). Furthermore, Zwolinski (2014) showed that, regarding well-being, previously excluded participants profited from another round of Cyberball in the inclusive version. So, it is likely that participants in the present study recovered even further, as they played Cyberball in the inclusive version one more time after the third measurement of emotions and needs (to measure approach/avoidance behavior). Nevertheless, the observed recovery was only in one of three indicators (negative affect) synchronously associated with decrease in goal desirability. Although this is consistent with the finding that goal disengagement capacities are significantly more strongly associated with negative (rather than positive) indicators of well-being (Barlow et al., 2020), the result must be interpreted cautiously due to the rather low effect size.

The exploratory analyses of the lagged correlations between change in goal desirability and change in well-being indicators further suggest that a reflexive drop in affect and need fulfillment in direct response to the goal blockage may be predictive of the strength of subsequent disengagement from the goal (in this case: decrease in well-being is associated with subsequent decrease in goal desirability). So, well-being indicators seem to be relevant here in the role of antecedents rather than outcomes of goal regulation processes. This fits with approaches in which affect is considered an important source of information for self-regulation (e.g., Carver, 2015; Forgas, 2017) and empirical findings that negative affect, particularly sadness, could promote goal disengagement (Koppe & Rothermund, 2016; Kunzmann et al., 2014).

In addition, the current study made first attempts to empirically measure another possible sub-process of cognitive-affective goal disengagement (change in relative group evaluation) as well as behavioral disengagement (approach/avoidance tendency towards “new” persons of the other group). Even though the effect of the behavioral approach/avoidance tendency was in the expected direction (participants in the blockage condition showed a preference for the new player over the one who had excluded one in the previous game while participants of the control condition showed no preference), there was no association of this behavioral goal disengagement with well-being at the end of the study nor with changes in goal desirability (i.e., a measure of previous cognitive-affective goal disengagement). Possibly, a preference for a new person over an old group member rather signals a “revenge” response indicating that the old belonging goal is still relevant. Complete disengagement (i.e., cognitive-affective as well as behavioral disengagement) might rather be marked by an equal ball-tossing distribution.

The unexpected results concerning the temporal dynamics of changes in relative group evaluation as another indicator of cognitive-affective goal disengagement could potentially be interpreted in the same way. Instead of the expected continuous relative devaluation of the group from which one was excluded, participants in the blockage condition first showed a very strong devaluation, which, however, decreased again somewhat in the regulation phase. In the above sense, the devaluation could be an expression of the current emotional state immediately after the exclusion. The regulation could consist of first perceiving this unpleasant state and then overcoming these strong negative feelings. The return to a rather “neutral” group evaluation would then represent regulation in progress (with complete disengagement, there should no longer be a negative attitude either, the group should be neutral again). This view is supported by the fact that both the relative devaluation of the own group from t1 to t2 and the subsequent partial revaluation from t2 to t3 were associated with an improvement in well-being from t2 to t3 (see Table 4) and thus seem to be functional.

## Limitations and future research

Regarding the regulatory function, only weak evidence could be found in the present study to suggest that disengagement from the goal to belong plays a functional role in restoring well-being after being excluded from playing Cyberball in a group. However, the various synchronous and asynchronous correlations point out the difficulty of separating antecedents, constituents, and consequences of goal disengagement processes if reciprocal influences over time are to be expected. Given that both onsets and durations of these processes are unknown, measurement at a higher temporal resolution is needed.

Moreover, the present findings are only based on self-report data and one behavioral indicator. Especially regarding the behavioral indicator, it is hard to differentiate between goal disengagement and reengagement processes. In the chosen implementation any decision not to pass a ball to a person (behavioral disengagement) also involves turning to another person. Thus, we cannot exclude that this behavior might also indicate reengagement in a new goal, at least for some participants. However, even if that were the case, a certain degree of behavioral disengagement or relative deprioritization is entailed in this kind of behavior, as behavioral reengagement and staying (behaviorally) committed are mutually exclusive in the given Cyberball task. Future studies could try to employ more sophisticated behavioral measures that allow the two processes to be differentiated.

Furthermore, the measurement of other facets of goal disengagement processes especially also on a sub-personal level is still a desideratum for future research. The procedure proposed in the present research provides a possible framework to develop and test such new measures. In particular, subsequent studies could attempt to measure cognitive-affective changes via sub-personal measures (for example, attentional changes associated with goal disengagement could be captured continuously via eye tracking, or changes in affective valence via neurophysiological measures). Traditional implicit measures (e.g., group evaluation via IAT; e.g., Pinter & Greenwald, 2011) could also be useful to record potential changes in attitudes and/or evaluations. Both ways could thus allow researchers to capture disengagement processes that are often assumed to take place quite unnoticed by the person who disengages and only become tangible and reportable for him or her in their outcome (“somehow this is no longer so important to me”). If measures at the sub-personal level are then combined with self-report measures, it could help to get a better insight into what is going on “under the surface” when disengaging from a goal.

Another aspect for future research concerns considering individual differences in the capacity to disengage. Previous theories and findings suggest that individual factors (such as dispositions or learning history) contribute to self-regulation and thus also to whether or not a person disengages from a blocked goal in a given situation (e.g., Burnette et al., 2013; Heckhausen & Wrosch, 2016). This is also reflected in the high variability of goal desirability change in the present study. However, in terms of the results of the study, this is a conservative problem because it increases variability within conditions, making effects harder to detect. At the same time, it indicates that once the effects can be replicated, the search for possible moderators might also be a fruitful endeavor for further studies. In this way, the procedure could also be used to experimentally vary possible situational influencing factors to gain a better understanding of what inhibits or promotes goal disengagement processes.

Lastly, it might be important to note that both studies reported here took place during the Covid-19 pandemic at a time when many contact restrictions were imposed. Especially for the younger generations, who made up the majority of study participants, many everyday opportunities to meet other people (such as school or university, sports clubs, and restaurants) were largely eliminated in Germany. Thus, it could be that the participants took part in the study in a quasi “socially deprived” state and that this reinforced both the induction of the goal to belong to the group and the effect of exclusion. Nevertheless, due to the high degree of agreement with findings from social psychology, whose stability has also been established in non-socially deprived participants, the existence of a cohort effect is rather unlikely.

## Conclusions

The present study combined with the pilot study forms a thorough first test of a procedure for the experimental study of goal disengagement processes based on Cyberball (Williams & Jarvis, 2006). Further studies are needed to replicate the effects. If the effects prove to be replicable, the procedure could be used in future studies to examine further processes assumed to constitute goal disengagement and develop other ways of capturing these, particularly sub-personal measures that are not based on self-report. The procedure also allows to systematically vary factors that are assumed to hinder or promote goal disengagement. Thus, the procedure presented here provides a basis for expanding research on goal disengagement processes, both in terms of a deeper causal understanding and in terms of intervention possibilities with practical relevance.

## Footnotes

1 Small deviations from the preregistration occurred only in the statistical analysis (contrast analysis and testing of hypothesis 10, for a detailed explanation, see Supplementary Material 1 uploaded on the OSF).

2 Hypotheses regarding the proposed design can be made from two perspectives: first, in terms of between-group comparisons, and second, in terms of intraindividual changes. Since individual regulation processes are the focus of the study, intraindividual changes, their predictors, outcomes, and relationships should play a central part. However, because previous studies (particularly those using the Cyberball paradigm in the context of ostracism research) have primarily used the procedure to induce or capture differences between people (and to implicitly infer intraindividual changes from them), hypotheses were made both in terms of intraindividual changes and between-group comparisons (concrete and more specific formulations addressing both perspectives and their correspondence to planned analyses can be found in the preregistration).

3 A detailed description of the procedure and materials, together with a list of all variables measured is uploaded on OSF.

4 A preliminary study was performed on ratings of these pseudonyms to ensure that they are as balanced and neutral as possible in terms of their perceptions (e.g., on their valence, familiarity and gender). Information on this study and data on the ratings of all 18 pseudonyms are available on request.

5 For procedures applied to ensure equal distribution of pseudonames, confederates, and session-specific influences across conditions, see Supplementary Material 2 on OSF.

6 A detailed report of the pilot study’s procedure, materials, further analyses and results is available at: https://osf.io/pcem9/.

7 We report uncorrected *p*-values in the results section, so that people who want to do so can use the desired adjustment method given multiple testing. In addition, we applied the conservative Bonferroni method to keep the family-wise type 1 error probability at 0.05, counting to a hypothesis family all tests applied under one of the numbered hypotheses for testing. Depending on the hypothesis, this ranges from one test (H1) to twelve tests (H3 and H7), so that the adjusted significance level for the individual tests in a hypothesis family ranged from .05 (H1) to .004 (H3 and H7) (for a detailed listing of the number of tests and adjusted significance levels by hypothesis family, see Supplementary Table 2 on OSF). Even with this conservative approach, there was almost no change in the decisions about the hypotheses (only in one case, which is pointed out in the results).

8 As the Shapiro-Wilk-test suggested deviations from normality, the more robust Wilcoxon-test was also performed, confirming the statistically significant difference (*p* < .001).

9 But, if, to control family-wise error rate, significance level is adjusted to .017 according to Bonferroni (as H8 formulates the same assumption for three indicators, 3 tests were performed that have to be considered: α = .05/3), this result would narrowly miss statistical significance.

10 Because the assumption of bivariate normality was not met in most cases, we used Spearman’s rho instead of Pearson’s *r* to calculate and test the strength of association.

## Declarations

### Open practices statement

The pilot study was not preregistered, the preregistration for the main study can be accessed at https://osf.io/2knfp. The materials used in these studies,further information of the procedure, supplemetary materials,  and anonymous data of both studies are available at https://osf.io/6k73g/.
